# Generation of BAC Transgenic Epithelial Organoids

**DOI:** 10.1371/journal.pone.0076871

**Published:** 2013-10-18

**Authors:** Gerald Schwank, Amanda Andersson-Rolf, Bon-Kyoung Koo, Nobuo Sasaki, Hans Clevers

**Affiliations:** 1 Hubrecht Institute, KNAW and University Medical Center Utrecht, Utrecht, The Netherlands; 2 Wellcome Trust - Medical Research Council Stem Cell Institute, University of Cambridge, Cambridge, United Kingdom; New York University, United States of America

## Abstract

Under previously developed culture conditions, mouse and human intestinal epithelia can be cultured and expanded over long periods. These so-called organoids recapitulate the three-dimensional architecture of the gut epithelium, and consist of all major intestinal cell types. One key advantage of these ex vivo cultures is their accessibility to live imaging. So far the establishment of transgenic fluorescent reporter organoids has required the generation of transgenic mice, a laborious and time-consuming process, which cannot be extended to human cultures. Here we present a transfection protocol that enables the generation of recombinant mouse and human reporter organoids using BAC (bacterial artificial chromosome) technology.

## Introduction and Results

In the past decades, the mouse has been extensively studied to understand vertebrate development. Progress has been largely driven by the generation of genetic tools, which enabled to manipulate the mouse genome. The generation of transgenic mice is however a time-consuming procedure, and many tissues are poorly accessible for in vivo live imaging.

We recently developed a method that allows the culture of three-dimensional multi-cellular structures from single Lgr5+ intestinal stem cells [1]. These so- called ‘miniguts’ recapitulating most features of the normal gut epithelium. Lgr5+ stem cells and the niche supporting Paneth cells are located in a domain that resembles the crypt bottom, and enterocytes as well as goblet - and enteroendocrine cells move upwards to build a villus-like domain that lines the central lumen. The organoid cultures are grown ex vivo in matrigel supplemented with a defined growth medium, and can be expanded for over a year. Direct genetic manipulation of organoid cultures has been previously demonstrated using a retroviral transduction based method, enabling overexpression and shRNA-mediated downregulation of target genes [2]. However, due to size-limitations of viral vectors entire genes including their cis-regulatory regions cannot be integrated into the host genome [3], and expression of transgenes under their endogenous promoter is therefore not possible. Here we present a method enabling stable insertions of more than 100 kilobase large BACs into mouse and human intestinal organoids, relying on liposome-based transfection.

**Figure 1:**
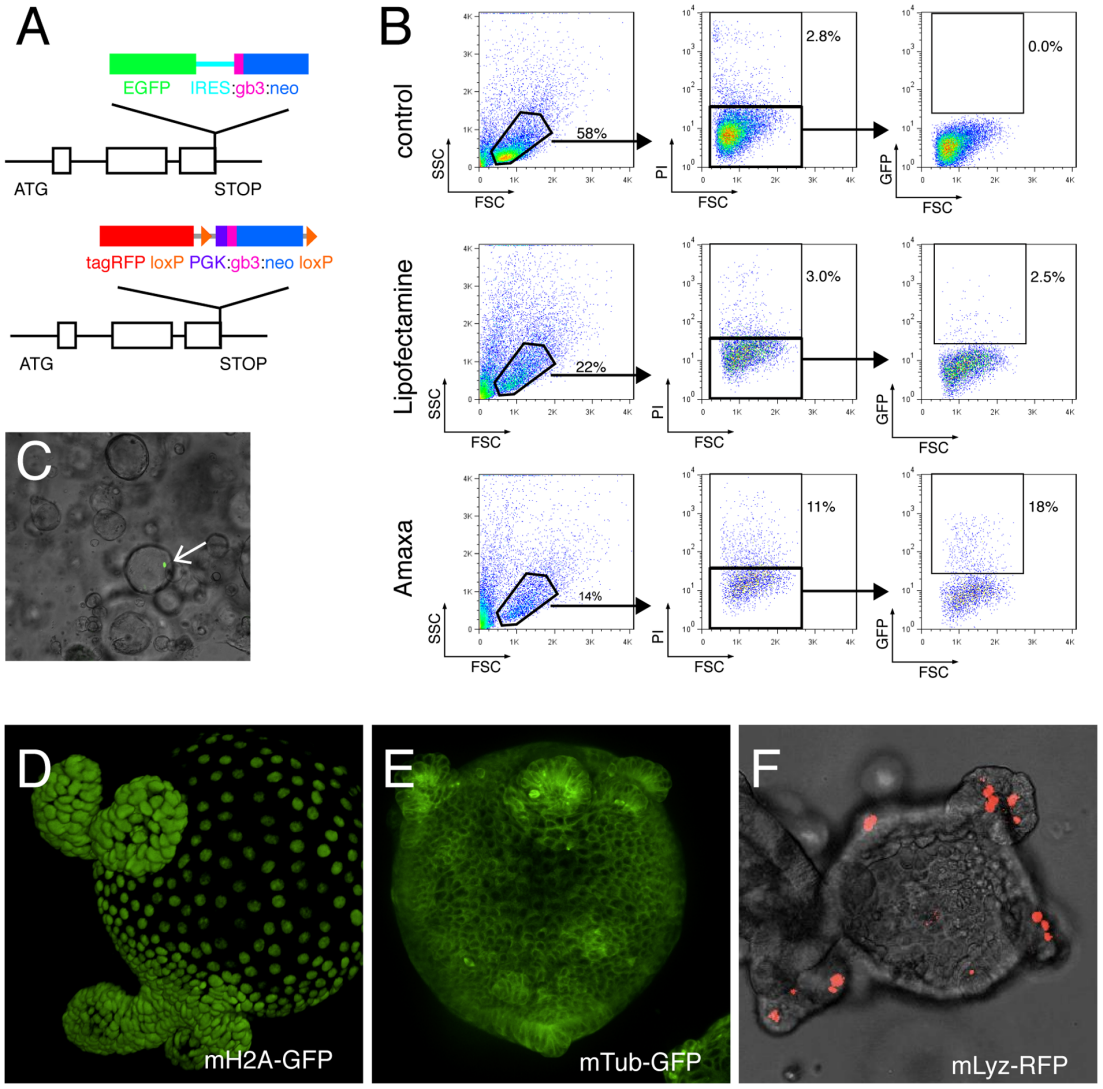
BAC transgenic mouse intestinal organoids. (A) Tagging cassettes for BAC recombineering. Upper panel illustrates the C-terminal GFP-tagging cassette with a neomycin resistance gene downstream of an IRES sequence, which was used in [7] to tag histone H2A and TUBB5. Lower panel shows the C-terminal tagRFP tagging cassette with a neomycine resistance gene downstream of a PGK promoter and flanked by loxP sites, which was used to tag the lysozyme gene. gb3: bacterial promoter, PGK: phosphoglycerate kinase promoter, IRES: internal ribosome entry site. (**B**) FACS analysis of mouse intestinal organoids 48 h after transfection with the pmax-GFP plasmid. Middle panel shows cell viability using propidium iodide (PI), right panel shows the percentage of GFP transfected cells. (**C**) Section of a well with mouse organoids 48 h after H2A-GFP BAC transfection using lipofectamine. Arrow points to a successfully transfected cell. Fluorescence images of a (**D**) H2A-GFP BAC transgenic organoid, (**E**) TUBB5-GFP BAC transgenic organoid, and (**F**) Lysozyme-tagRFP BAC transgenic organoid.

Due to their large size BACs are able to carry the entire genomic locus of genes, and therefore often ensure precise expression patterns [4]. BAC libraries covering the entire mouse and human genome have been established (www.chori.org), and BAC recombineering allowed the generation of libraries with fluorescently tagged genes [5], [6]. Here we used previously established BAC reporters with enhanced green fluorescent (EGFP) tagged genes [7], and in addition generated recombineering cassettes that allow protein tagging with the red fluorescent protein tagRFP [8], the cyan fluorescent protein mTurquoise [9], and the yellow fluorescent protein mVenus [10] (Fig. 1A). These vectors carry a kanamycin-neomycin selection marker, which is flanked by loxP sites and therefore enables excision using Cre recombinase [11]. The tamoxifen inducible *CreERT2* can be delivered to mouse and human organoids by retroviral infection, or by deriving organoids from transgenic mice expressing the enzyme [2], [12].

To explore methods for transfection of mouse intestinal organoids we delivered a 3.5 kb reporter plasmid expressing EGFP (pmax-GFP, Amaxa™) into organoids, using either electroporation based transfection (Nucleofector™) or liposome-mediated transfection (Lipofectamine®). We expanded mouse intestinal organoids in Wnt-conditioned media to enrich for stem cells, separated them from matrigel by pipetting, and trypsinized them in order to get a single cell suspension. Cells were then transfected as described in Materials and Methods, and analyzed for EGFP expression 48 hours later. Nucleofection lead to a transfection efficiency of 18%, and lipofectamine-mediated transfection resulted in 2.5% positive cells (Fig. 1B). We next transfected mouse organoids with a 120 kb BAC reporter containing the genomic locus of the core histone H2A with a C-terminal EGFP tag [7] (Fig. 1A). While nucleofection did not lead to successful BAC transfection, lipofection resulted on average in 3.8 (+/−1.3 STD) positive cells per well (Fig. 1C). Co-expression of an IRES-driven neomycin resistance gene enabled selection for stable expression. After two weeks organoids showed uniform nuclear GFP localization (Fig. 1D), and expression remained stable for more than 6 months. Similarly, we were able to transfect a BAC reporter containing the EGFP-tagged genomic locus of the ß-Tubulin gene TUBB5, a major constituent of microtubules. After selection, organoids ubiquitously expressed the transgene and had a GFP labeled microtubule network, including the mitotic spindle of dividing cells (Fig. 1E).

Next, we tested if our method can be used to label specific cell lineages. We generated a BAC reporter with tagRFP labeled lysozyme, a specific marker for Paneth cells [13], and transfected organoids from *villin-creERT* transgenic mice [12]. IRES could not be used to drive the neomycin resistance gene, as the lack of lysozyme expression in stem cells would kill also successfully transfected organoids. Instead we used the constitutively active PGK promoter (Fig. 1A). After selection and CreERT2-mediated removal of the neomycin selection cassette, recombinant organoids specifically expressed the tagRFP reporter in Paneth cells located at the base of the organoid crypts (Fig. 1F).

**Figure 2:**
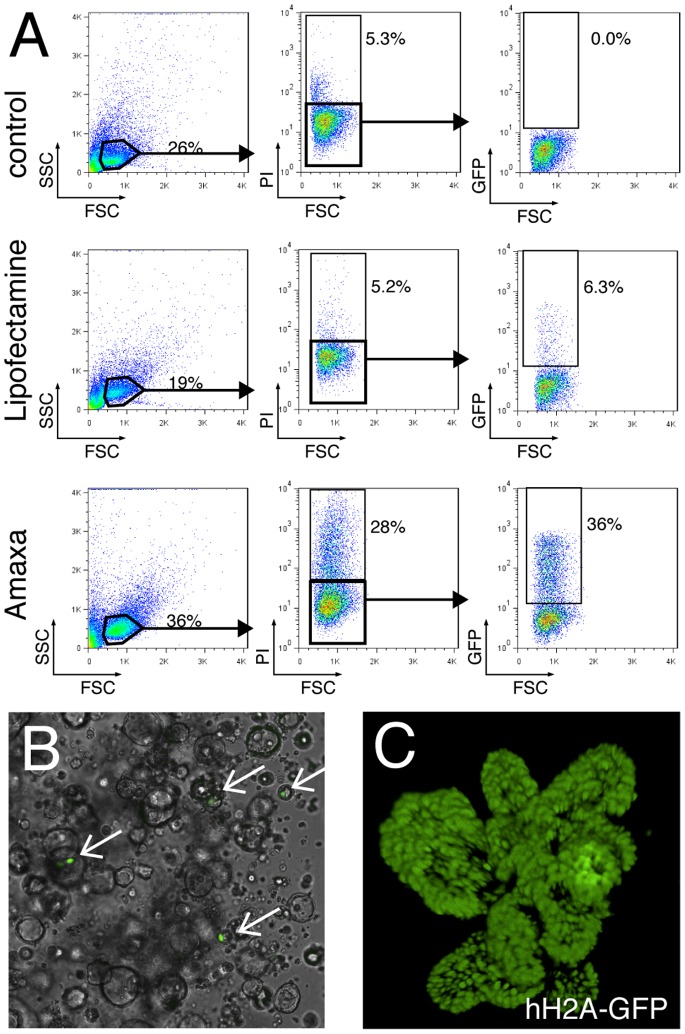
BAC transgenic human intestinal organoids. (**A**) FACS analysis of human intestinal organoids 48 h after transfection with the pmax-GFP plasmid. Middle panel shows cell viability using propidium iodide (PI), right panel shows the percentage of GFP transfected cells. (**B**) Section of a well with human organoids 48 h after H2A-GFP BAC transfection with lipofectamine. Arrows point to successfully transfected cells. (**C**) Fluorescence image of a H2A-GFP BAC transgenic human organoid.

Modification of the original growth-factor composition has allowed us to also grow epithelial organoids derived from human intestine [14], [15]. To test if our method can be applied to human organoid cultures, we optimized the protocol and transfected human small intestinal organoids with the pmax-GFP plasmid. Surprisingly, both transfection methods were more efficient in human organoids compared to mouse organoids; nucleofection lead to a transfection efficiency of 36%, and lipofectamine-mediated transfection resulted in 6.3% positive cells (Fig. 2A). We next used lipofectamine to transfect human organoids with the histone H2A-GFP BAC reporter, resulting on average in 36.6 (+/−6.3 STD) positive cells per well (Fig. 2B). Stable clones were selected using G418 and ubiquitously expressed nuclear H2A-GFP (Fig. 2C).

**Figure 3:**
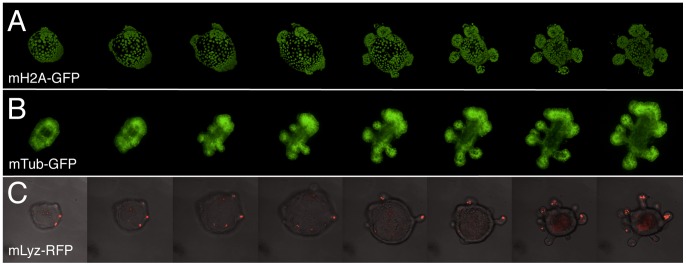
Snapshots of BAC transgenic organoid movies. (**A**) a 36 h movie (2.4 frames/hour) of a growing mouse H2A BAC transgenic organoid, (**B**) a 51 h movie (2.2 frames/hour) of a growing mouse TUBB5-GFP BAC transgenic organoid, and (**C**) a 77 h movie (1 frame/hour) of a growing mouse Lysozyme-tagRFP BAC transgenic organoid.

As a proof of concept we performed time lapse ex vivo imaging on the BAC transgenic organoid lines. We used conventional confocal and spinning disc microscopes to image organoids up to 77 hours ((Fig. 3), Movies S1–S3). The 3D reconstruction of the acquired images allowed us to visualize spatiotemporal processes such as crypt bud formation, cell division, and growth (Movies S1–S3).

Taken together, we present a method that allows the generation of BAC recombinant organoids. This technique can be used to study gene function ex vivo in organoid cultures under endogenous expression levels by live imaging, and is applicable to mouse and human organoid cultures. Thus, it will help to circumvent the time-consuming and costly process of generating transgenic mice, and enable to study the role of genes in human epithelial tissues, potentially opening a new avenue to examine genes involved in human diseases.

## Materials and Methods

### Human Samples

This study was approved by the ethical committee of the University Medical Centre Utrecht, and all samples were obtained with informed consent. The participants provided their informed consent to participate in this study in a written form.

### Mouse Samples

All animal experiments have been conducted according to relevant national and international guidelines. Experimental setup was approved by the animal welfare committee (DEC) of the Royal Dutch academy of sciences (KNAW).

### Organoid Culture

Crypts were isolated from mouse and human small intestines by incubating the tissue for 60 minutes with 2 mM EDTA in PBS at 4°C. Detached crypts were subsequently plated in 20 µl drops of matrigel, and after polymerization the previously described growth medium was added [16]. In short, mouse intestinal growth medium consists of advanced DMEM/F12 medium (Invitrogen) including the supplements B27 (Invitrogen), N2 (Invitrogen) and N-Acetylcysteine (Sigma-Aldrich) and the growth factors noggin (Peprotech), Rspo1 [17], and epidermal growth factor (Peprotech). Human intestinal growth medium additionally contains Wnt conditioned media, TGF-ß type I Receptor inhibitor A83-01 (Tocris), Nicotinamide (Sigma-Aldrich) and P38 inhibitor SB202190 (Sigma-Aldrich). Confluent organoids were mechanically dissociated using a fire polished glass pipette. Fragmented organoids were centrifuged at 1000 *g* for 5 minutes, and resuspended in cold matrigel in a 1∶4 ratio. Rosa-CreERT2 mice were used to generate mouse small intestinal organoids. Isolation of human crypts was described elsewhere [15].

### Vector Construction

BAC recombineering cassettes: For cloning we used the In-Fusion Advanced PCR cloning kit (Clontech). The *loxP-pgk:gb2:neo-loxP* cassette was PCR amplified (with the 5′ primer containing a NotI site and the 3′ primer containing a XhoI site) from the R6Kamp-hNGFP vector (kindly provided by Anthony Hyman, MPI Dresden), and inserted into the HspI site of the pMSCV puro vector (Clontech). Vectors containing the fluorescent proteins mTurquoise and mVenus were kindly provided by Joachim Goedhart (University of Amsterdam), and tagRFP was obtained by Evrogen. The sequences for the fluorescent proteins were PCR amplified with primers that contain a nuclear localization sequence after the start codon, and cloned upstream of the *loxPpgk:gb2:neoloxP* cassette into the NotI restriction site. The BGH polyA signal (Invitrogen) was PCR amplified and cloned downstream of the *loxP-pgk:gb2:neo-loxP* cassette into the XhoI restriction site. Additionally we also generated vectors with the SV40 polyA signal (PCR amplified from the pTagRFP-N vector (Evrogen)) between the fluorescent protein sequences and the *loxP-pgk:gb2:neo-loxP* cassette. For BAC recombineering a 360 bp homology region around the lysozyme stop codon was synthesized and cloned into pIDTsmart backbone (IDT). The sequence was flanked with SacII sites, and the regions upstream and downstream of the stop codon were separated by BamHI and XbaI sites. This design allowed to insert the *tagRFP;loxP-pgk:gb2:neo-loxP* cassette between the homology arms, and to linearize the entire construct for recombineering.

### BAC Recombineering

The BAC clone RP11-1105J23 which contains the Lysozyme 2 locus (ENSMUSG00000069516) was obtained from the BACPAC resources center (Children’s Hospital Oakland Research Institute in Oakland, California, USA). Recombineering was done using the Quick and Easy BAC Modification Kit (Gene Bridges), and the provided protocol was followed. The recombineering cassette was linearized using the SacII sites and purified by LiCl precipitation. 0.5 µg of DNA were used for the transfection. H2A-GFP (ENSMUSG00000037894) and tubb5-GFP (ENSMUSG00000001525) BACs were kindly provided by Anthony Hyman (MPI Dresden).

### Preparing Organoids for Transfection

Before transfection mouse organoids were cultured for two generations in growth medium plus Nicotinamide and Wnt-conditioned medium. Under these conditions the cultures mainly consist of stem cells, which can form new organoids after seeding single cells. Stem cell enriched organoids were first mechanically dissociated (per transfection reaction organoids of approximately six 20 µl matrigel drops were used), transferred into 15 ml falcon tubes and centrifuged for 5 minutes at 1000 *g*. The pellet was resuspended in TriplE (Invitrogen) and trypsinized for 5 minutes at 37°C to obtain single cells. Human organoids were grown in expansion media, which already contains Wnt and Nicotinamide (see above). To obtain single cells, human organoids were trypsinized in TriplE for 10–15 minutes at 37°C, with short vortexing steps every 3 minutes.

### Transfection using Electroporation

We used the Amaxa™ Mouse/Rat–Hepatocyte–Nucleofector™ kit. After trypsinization cells were spinned at 1000 *g* for 5 minutes and the pellet was resuspended in nucleofactor solution plus supplement and plasmid DNA. Electroporation was performed according to the standard Amaxa protocol. After electroporation cells were incubated for 15 minutes at room temperature in the nucleofactor solution. The cell suspension was transferred to an Eppendorf tube, spun at 1000 *g* for 5 minutes, resuspended in 100 µl cold matrigel, and split into 5 wells of a 48-well culture plate. After polymerization we added growth medium plus Nicotinamide, Wnt-conditioned medium, and the Rho kinase inhibitor Y-27632 to mouse organoids. To human organoids we added human expansion media plus Y-27632.

### Transfection using Liposomes

After trypsinization cells were spun at 1000 *g* for 5 minutes, the supernatant was removed, and mouse cells were resuspended in 450 µl growth medium plus Nicotinamide, Wnt-conditioned media and the Rho kinase inhibitor Y-27632 (human cells were resuspended in human expansion media plus Y-27632). Cells were then plated in 48 well plates at high density (80–90% confluent). Nucleic acid-Lipofectamine® 2000 complexes were prepared according to the standard Lipofectamine protocol. In short, 4 µl of Lipofectamine® 2000 reagent and 1.5 µg plasmid DNA were each diluted in 50 µl Opti-MEM® medium. Both mixes were pooled and incubated for 5 minutes before the DNA-reagent complex was added to the cells (50 µl per well). We centrifuged the plate at 600 *g* at 32°C for 60 min, and then incubated the plate for additional 4 hours at 37°C. Cells were collected in eppendorf tubes, centrifuged at 1000 *g*, resuspended in 100 µl cold matrigel, and plated as described above in the electroporation protocol.

### After Transfection

Two days after transfection we added 200 µg/ml G418 (Invitrogen) to the medium. When necessary organoids were split 1∶3. After selection of stable organoids Wnt3a and Nicotinamid were removed from the mouse organoid media, and sphere-like organoids changed into budding organoids within 1–2 weeks. For the cell sorting experiments organoids were trypsinized 48 h after the transfection, and single cells were analyzed using a MoFlo (Dako Colorado, Inc.) FACS machine.

### Live Imaging and Image Analysis

Images and movies of histone H2A-GFP and tubulin TUBB5-GFP organoids were taken with a PerkinElmer Ultraview VoX spinning disk microscope. For image analysis the Volocity 3D image analysis software (PerkinElmer) was used. Lysozyme-tagRFP organoids were imaged using a Leica Sp5 confocal microscope. For image analysis ImageJ was used.

## Supporting Information

Movie S1 **36 h movie (2.4 frames/hour) of a growing mouse histone H2A BAC transgenic organoid.**
(M4V)

Movie S2 **51 h movie (2.2 frames/hour) of a growing mouse tubulin TUBB5-GFP BAC transgenic organoid.**
(M4V)

Movie S3 **77 h movie (1 frame/hour) of a growing mouse Lysozyme-tagRFP BAC transgenic organoid.**
(AVI)
